# Long-term drinking behavior change patterns and its association with hyperuricemia in chinese adults: evidence from China Health and Nutrition Survey

**DOI:** 10.1186/s12889-022-13637-4

**Published:** 2022-06-20

**Authors:** Bowen Zhu, Yang Li, Yiqin Shi, Nana Song, Yi Fang, Xiaoqiang Ding

**Affiliations:** 1 Department of Nephrology, Zhongshan Hospital, Fudan University, No.180 Fenglin Road, 200032 Shanghai, Shanghai China; 2 Shanghai Medical Center of Kidney, Shanghai, China; 3 Shanghai Key Laboratory of Kidney and Blood Purification, Shanghai, China

**Keywords:** Hyperuricemia, Drinking behavior change patterns, Alcohol consumption, China Health and Nutrition Survey, Nutritional epidemiology

## Abstract

**Background:**

We aimed to explore the association between long-term drinking behavior change patterns with hyperuricemia (HUA) in Chinese community adults.

**Methods:**

This study was designed as a community-based unbalanced cohort study involving 4127 adults aged between 18 ~ 75 years, derived from the China Health and Nutrition Survey (CHNS) in 1997 and 2009. Drinking behavior change patterns were categorized into: never drinking, change to drinking, quitting drinking, and continued drinking. The alcoholism, type, and frequency of drinking were further categorized. We applied logistic regression models to explore the associations between drinking behavior change patterns and HUA.

**Results:**

The average age of the participants was 54.6 (± 11.3) years and 47.8% were male. The overall prevalence of HUA was 15.5%. Drinking behavior change patterns of quitting (aOR 1.8; 95% CI 1.1 ~ 2.8) and continued drinking (aOR 2.0; 95% CI 1.3 ~ 3.0) were positively associated with high risks of HUA in the male participants. Early drinking behaviors such as liquor intake (aOR 1.8; 95% CI 1.4 ~ 2.5) and high consumption or frequency showed a positive correlation with HUA. Of note, heavy alcoholism (aOR 2.0; 95% CI 1.4 ~ 2.8) and daily drinking (aOR 2.5; 95% CI 1.7 ~ 3.6) had the highest risks of HUA. Furthermore, in the male participants, the association between early total alcohol intake and HUA was more pronounced at 18 standard drinks intake, with a stable increasing trend. In contrast, no statistical correlation was observed between the drinking behaviors and HUA in the female participants.

**Conclusions:**

Drinking behavior change patterns of quitting and continued drinking are strongly associated with increased risks of HUA in males. The risks emanated from early drinking behaviors such as liquor drinking, high drinking frequency, and alcohol consumption. Although quitting drinking was associated with lower HUA risks compared to continued drinking, it still presented an undeniable risk for HUA.

**Supplementary Information:**

The online version contains supplementary material available at 10.1186/s12889-022-13637-4.

## Introduction

Hyperuricemia (HUA) is a potentially modifiable risk factor for kidney dysfunction, cardiovascular disease (CVD) or death, and affects 21% of the world population [1–3]. The burden of HUA has dramatically increased over recent decades: from approximately 8.5% in 2001 to approximately 18.4% in 2017 in China [4]. Asymptomatic HUA is associated with daily routine lifestyle activities, such as regular exercise, smoking status, daily diet structure, or alcohol drinking behaviors [5]. Notably, there are specific drinking patterns, demographic, physical indicators, or distribution in the Chinese population. The risks of drinking and associated behaviors in China have constantly changed over generations from 1993 to 2011, and are likely to continue through 2027 in China [6]. The China Kadoorie Biobank reported that 8% of males are drinkers and individuals engaging in heavy drinking episodes were likely to have multiple risk factors such as regular smoking, low physical activity, and hypertension [7]. Various diseases including diabetes, chronic kidney disease (CKD), and ischemic stroke are associated with heavy alcohol intake in the Chinese population [8, 9]. Although drinking behaviors vary over time in China, there is emerging evidence that defines the potential correlation of the changing drinking behaviors with the development of HUA.

Several cohort studies have reported that alcohol drinking is associated with approximately 1.5 ~ 2.0 fold higher risk with HUA, compared with non-drinking individuals [10]. The risk of HUA could conceivably vary depending on the type of alcoholic beverage (i.e., beer, wine, and liqueur) or alcohol consumption [11]. A prospective study reported that alcohol consumption is strongly associated with increased risk of gout with a linear trend, and beer confers a higher risk than spirits, whereas moderate wine drinking does not fuel the risk [12]. Currently, data on the effect of the change in drinking behaviors on HUA as well as the underlying reasons remain scanty. Besides, most of these studies did not quantify standard alcohol intake.

To provide scientific evidence for the long-term alcohol consumption change patterns and their association with the risk of HUA among Chinese, data from the China Health and Nutrition Survey (CHNS) was used to explore the effect of long-term alcohol change patterns between 1997 and 2009 on the risk of HUA. The data on the risks of HUA and its association with different drinking patterns over time would have novel implications on the prevention and management of HUA in the Chinese population.

## Materials and methods

### Study design

CHNS was an ongoing cohort from 1989 to 2015 up to now, as well as an international collaborative project at the Chinese Center for Disease Control and Prevention (CCDC). The CHNS data included nine provinces (Liaoning, Jiangsu, Shandong, Henan, Hubei, Hunan, Guangxi, Guizhou, and Heilongjiang). It aims to characterize how the social and economic transformation of Chinese society against the health and nutritional status of its population [13]. Since data on biomarkers including serum uric acid (SUA) were firstly performed in 2009 and alcohol consumption information was systematically collected in 1997, we extracted the data between 1997 and 2009. Overall, the data in our study was unbalanced longitudinal data structure with two drinking-related behaviors measurements and one SUA measurement. A total of 5335 individuals with alcohol consumption data and biomarkers matched by ID (marked as idind) were obtained from the surveys. Our study population consisted 14,052 participants in the 1997 survey wave, who provided demographic, physical examination and health behavioral-related information. Of them, 5335 participants with blood biomarkers information were followed up in 2009. After applying the exclusion criteria (Supplementary Fig. 1), a total of 4127 participants were included in the formal analysis.

### Data collection

A standardized structured questionnaire was administered by trained health staff to collect socio-demographic variables such as age, sex, educational attainment, urban-rural residence, history of diseases (hypertension, diabetes, apoplexy, and myocardial infarction), smoking status, alcohol use, tea intake, coffee intake, total protein intake, and extent of physical activity level. Measurement of waist and hip circumference, height, weight, and blood pressures (BP) were performed by trained clinical staff [14]. All individuals maintained a regular life pattern for at least three days before blood sample collection and 12 ml of blood was collected (in three 4 ml tubes) on empty stomach (http://www.cpc.unc.edu/projects /china/data/datasets/ biomarker-data). The biomarker data collected from CHNS in 2009 involved 26 fasting blood parameters on individuals over 7 years old [14]. Plasma and serum samples were then frozen and stored at -86 °C for later laboratory analysis. All samples were assayed in a national central lab in Beijing (medical laboratory accreditation certificate ISO 15189:2007) with strict quality control.

Total cholesterol (TC) was assayed using the CHOD-PAP (Hitachi 7600, Kyowa, Japan). Low-density lipoprotein cholesterol (LDL) was assayed using the enzymatic method (Hitachi 7600, Kyowa, Japan). Triglyceride (TG) was assayed using the GPO-PAP (Hitachi 7600, Kyowa, Japan). Creatinine was assayed using the picric acid method (Hitachi 7600, Randox, UK). The data were available online: https://www.cpc. unc.edu/projects/ china.

Data on educational year was derived from the questionnaires and divided into five categories: 0, 6 years or less, 6–8 years, 9–11 years, and 12 years or higher. Living conditions were divided into urban and rural. Smoking status was assessed by the question including ‘Ever smoked cigarettes?’ or ‘Still smokes cigarettes?’, with three response options: ‘no’, ‘yes’ or ‘unknown’. The smoking status was categorized as non-smokers, ex-smokers, and current smokers. The total Metabolic equivalent (MET) per week was calculated to quantify the extent of physical activities. It was a composite index calculated by multiplying the frequency, duration, and intensity of physical activity, and categorized into tertiles [15]. Individual dietary intake for 3 consecutive days was determined for every household member. This determination was achieved by asking individuals to report all food consumed at home or away from home on a 24-hour recall basis each day. Body mass index (BMI) was calculated as weight in kilograms divided by the square of height in meters (kg/m^2^). The BMI was then categorized into four levels: lean (< 18.5 kg/m2), normal (18.5 ~ 23.9 kg/m^2^) or overweight (24.0 ~ 27.9 kg/m^2^) and obesity (≥ 28 kg/m^2^). The waist-to-hip ratio (WHR) was calculated as waist circumference (cm)/height (cm). The cutoffs for the WHR were set at 0.9 for men and 0.85 for women, according to the World Health Organization (WHO) guidelines [16]. The average SUA was recorded and HUA was defined as ≥ 7 mg/dL for males or 6 mg/dL for females [17]. The systolic and diastolic BP were expressed as a mean of three measurements. Hypertension was defined by a systolic BP ≥ 140 mmHg or diastolic BP ≥ 90 mmHg or self-reported by questionnaire [18]. Diabetes mellitus was self-reported or obtained from diabetes treatment records. Dyslipidaemia was defined as total cholesterol 5.2 mmol/L or higher, LDL cholesterol 3.4 mmol/L or higher, or triglycerides 1.7 mmol/L or higher [19]. Estimated glomerular filtration rate (eGFR) was calculated as chronic kidney disease epidemiology collaboration (CKD-EPI) 2009 creatinine equation [20].

### Alcohol consumption change patterns

Drinking behaviors were assessed through the question: ‘Have you ever had beer, liquor or other alcoholic beverages?’, and three responses were sought: ‘no’, ‘yes’ or ‘unknown’. Alcohol drinkers were further asked to report the drinking frequency, types, and average weekly beer consumption (bottles/week), wine (grams/week), and liquor (grams/week). The drinking frequency was defined as never (no drinking), less than weekly (< 1 time/week), weekly (1–4 times/week), or daily (almost every day). The alcohol concentration in different alcoholic beverages was in accordance with the 2010 China monitoring report on chronic disease risk factors (beer = 4%, grape wine = 10% and liqueur = 38%) : 1 bottle = 600 ml, 1 Liang = 50 ml [21]. A calculation method was provided for the volume of alcohol contained in the beverages and a formula to estimate the total volume of alcohol consumed:

A Alcohol intake (beer) = bottle * 600 ml * 0.04.
B Alcohol intake (grape wine) = Liang * 50 ml * 0.1.
C Alcohol intake (liqueur) = Liang * 50 ml * 0.38.

Total alcohol intake (Standard Drinks [SD])= (A + B + C)/10 g*0.789.

The alcoholism was divided into none (no drinking), mild (total alcohol intake < 14 SDs per week for men or < 7 SDs per week for women), or heavy (total alcohol intake ≥ 14 SDs per week for men or ≥ 7 SDs per week for women) [22]. Alcohol change patterns were assessed based on the current alcohol drinking (in 2009) and baseline alcohol drinking (in 1997). Drinking behavior change patterns were categorized into: never drinking (not drinking in 1997 and not drinking in 2009), change to drinking (not drinking in 1997 and drinking in 2009), quitting drinking (drinking in 1997 and not drinking in 2009), and continued drinking (drinking in 1997 and drinking in 2009). Combined with the total alcohol intake and alcohol change patterns, alcohol change patterns were further divided into never drinking, abstainer to mild, abstainer to heavy, mild to abstainer, mild to mild, mild to heavy, heavy to abstainer, heavy to mild, heavy to heavy. The type of drinking was categorized into beer drinker, wine drinker (including fruit wine, yellow rice wine, rice wine, etc.), or liquor drinker. In addition, the drinking frequency was categorized into no drinking, less than weekly, weekly, or daily.

### Statistical analysis

Data were presented as mean ± standard deviation (SD) for continuous variables or N (%) for categorical variables. Group comparison of drinking behaviors was performed using the chi-square test, fisher’s exact test for categorical variables, and variance analysis for continuous variables where appropriate. Univariable and multivariable logistic regression models were used to explore the association between demographic, anthropometry, biochemical index or behavior information, and HUA. To determine whether drinking behavior change patterns and early drinking behaviors were independently associated with the HUA by gender. Variables that were both associated with the HUA and deemed to be causally related to drinking-related behaviors were included as potential confounders (Supplementary Table 3). Multivariable logistic models were sequentially adjusted for: age (as continuous), BMI, WHR, hypertension, and diabetes; and smoking status, total protein intake (as continuous). Characteristics in the analytic sample and excluded samples were compared to explore potential selection bias on study results. Multivariate logistic regression analysis was performed to assess the dose-response correlation between alcohol intake and HUA by raising the alcohol intake cutoff point from 2 to 30 SDs for both males and females. Prespecified subgroup analyses were conducted to explore the association between drinking behavior change patterns and early drinking behaviors with HUA based on gender and age (18 ~ 29, 30 ~ 44, 45 ~ 59, 60 ~ 75 years) group. To determine the longitudinal associations of drinking behavior change patterns and drinking-related behaviors with HUA, GEE analyses were performed. The results were presented as odds ratios (OR) with 95% confidence intervals (95% CI). A two-sided *p*-value < 0.05 was used as a threshold of statistical significance. Data were analyzed using SAS version 9.3 (SAS Institute Inc).

## Result

### Characteristics of participants

 A total of 4462 participants aged between 18 and 75 in 1997 were recruited at the beginning of our study. Out of the total 335 participants were excluded because 6 were pregnant, 211 had average protein dietary intake for three consecutive days of > 110 g/day, 25 had the end-stage renal disease (ESRD) while 93 had no data on SUA. Finally, 4127 participants were included in the formal analysis (Supplementary Fig. 1). Demographic and behavioral characteristics of the analytic sample and excluded samples with missing SUA data were compared (Supplementary Table 1). Additionally, demographic and behavioral characteristics of the follow-up sample (*n* = 5335) and missing visits sample (*n* = 8717) were compared. Follow-up sample had higher proportion of old age, obese WHR, BMI ≥ 24 kg/m^2^, diabetes and hypertension (Supplementary Table 2). The findings indicated that most of the characteristics had no significant differences (P > 0.05). The average age of participants was 54.6 (± 11.3) years and 47.8% (1974/4127) were male. The overall prevalence for HUA was 15.5%. Of the 4127 participants, 53.0% never drunk, 10.3% changed to drinking, 12.9% quit drinking while 23.8% continued drinking. Individuals who continued drinking were more likely to be current smokers, have higher education and have higher CVD risk factors. On the contrary, individuals who quit drinking had more traditional risk factors such as old age, hypertension, history of apoplexy (Table 1).

**Table 1:** Characteristics of participants among four groups of drinking behavior change patterns (*n* = 4127)

	Drinking behavior change pattern				Total	P-value*
	Never drinking	Change to be drinkers	Quit drinking	Keep drinking		
Participants (n)	2187	424	532	984	4127	
Age (years)	54.5 (± 11.1)	49.4 (± 12.2)	56.8 (± 11.1)	53.8 (± 10.4)	54.6 (± 11.3)	< 0.001
Male (%)	369 (16.9)	302 (71.2)	368 (69.2)	935 (95.0)	1974 (47.8)	< 0.001
Education (years)						< 0.001
0	475 (21.7)	42 (9.9)	64 (12.1)	54 (5.5)	635 (15.4)	
1–6	805 (36.8)	122 (28.8)	187 (35.0)	311 (31.6)	1425 (34.5)	
7–9	607 (27.7)	171 (40.2)	176 (33.2)	376 (38.3)	1330 (32.1)	
10–12	180 (8.2)	47 (11.1)	60 (11.3)	143 (14.6)	430 (10.4)	
> 12	120 (5.5)	42 (9.9)	45 (8.5)	100 (10.2)	307 (7.4)	
Rural (%)	1634 (74.7)	288 (67.9)	367 (69.0)	702 (71.3)	2991 (72.5)	0.003
*Anthropometry parameters*						
Waist (cm)	82 (± 10)	82 (± 10)	84 (± 10)	85 (± 10)	83 (± 10)	< 0.001
Hip (cm)	94 (± 8)	94 (± 7)	94 (± 8)	94 (± 8)	94 (± 8)	0.531
Obese WHR	1155 (54.4)	177 (42.9)	233 (45.5)	457 (48.3)	2022 (49.0)	< 0.001
BMI (kg/m^2^)						0.002
Lean (< 18.5)	129 (5.9)	26 (6.1)	22 (4.1)	41 (4.2)	218 (5.3)	
Normal (18.5–23.9)	1185 (54.2)	236 (55.7)	303 (57.0)	543 (55.2)	2267 (54.9)	
Overweight (24–27.9)	648 (29.6)	120 (28.3)	159 (29.9)	340 (34.6)	1267 (30.7)	
Obesity (≥ 28.0)	225 (10.3)	42 (9.9)	48 (9.0)	60 (6.1)	375 (9.1)	
Systolic BP (mm Hg)	125 (± 19)	122 (± 17)	126 (± 18)	126 (± 17)	125 (± 19)	0.010
Diastolic BP (mm Hg)	80 (± 11)	80 (± 11)	81 (± 10)	83 (± 12)	81 (± 11)	< 0.001
Hypertension	557 (29.4)	86 (22.7)	149 (34.1)	244 (29.6)	1036 (25.1)	0.005
Diabetes	56 (2.6)	5 (1.2)	19 (3.6)	25 (2.5)	105 (2.5)	0.142
Serum uric acid (mg/dL)	5 (± 2)	5 (± 2)	5 (± 2)	6 (± 2)	5 (± 2)	< 0.001
Hyperuricemia	258 (11.8)	68 (16.0)	92 (17.3)	222 (22.6)	640 (15.5)	< 0.001
Dyslipidemia	1314 (60.1)	236 (55.7)	327 (61.5)	619 (62.9)	2496 (60.5)	0.075
eGFR (ml/ min/l.73m^2^)	68 (± 18)	83 (± 33)	79 (± 15)	83 (± 14)	81 (± 18)	< 0.001
History of MI	22 (1.0)	3 (0.7)	9 (1.7)	6 (0.6)	40 (1.0)	0.208
History of apoplexy	16 (0.7)	3 (0.7)	14 (2.6)	8 (0.8)	41 (1.0)	< 0.001
*Health-related behavior*						
Smoking status						< 0.001
Never	1929 (88.2)	206 (48.6)	335 (63.0)	311 (31.6)	2781 (67.4)	
Ever	33 (1.5)	27 (6.4)	31 (5.8)	40 (4.1)	131 (3.2)	
Current	224 (10.3)	191 (45.1)	166 (31.2)	633 (64.3)	1214 (29.4)	
Tea intake	600 (27.4)	212 (50.0)	187 (35.2)	493 (50.1)	1492 (36.2)	< 0.001
Coffee intake	25 (1.2)	16 (3.8)	7 (1.3)	18 (1.8)	66 (1.6)	0.001
Total protein intake (g/day)	59 (± 18)	68 (± 18)	63 (± 19)	68 (± 18)	63 (± 19)	< 0.001
Physical activity level (METs/week)						0.870
Low (< 49.6)	728 (33.3)	130 (30.7)	185 (34.8)	333 (33.8)	1376 (33.3)	
Medium (49.6 ~ 143.7)	729 (33.3)	150 (35.4)	168 (31.6)	328 (33.3)	1375 (33.3)	
High (> 143.7)	730 (33.4)	144 (34.0)	179 (33.7)	323 (32.8)	1376 (33.3)	

*Abbreviation: BMI *body mass index, *BP *blood pressure, *eGFR *estimated glomerular filtration rate, *MI *myocardial infarction, *SUA *serum uric acid, *WHR *waist to hip circumference ratio. Data are presented as No. (%), mean ± SD or median (IQR)

*P values were calculated by using T-test or Wilcoxon test for continuous variables and χ2 test or Fisher exact test for categorical variables

133 participants were not available for WHR; 591 participants were not available for hypertension; 31 participants were not available for drinking frequency; 9 participants were not available for coffee intake; 7 participants were not available for tea intake; 3 participants were not available for the history of myocardial infarction; 1 participant was not available for the history of apoplexy; 1 participant was not available for smoking status; 26 participants were not available for drinking frequency; 9 participants were not available for beer drinking; 10 participants were not available for wine drinking; 11 participants were not available for liquor drinking

### Prevalence and association of drinking behavior change patterns with HUA

**Fig. 1:**
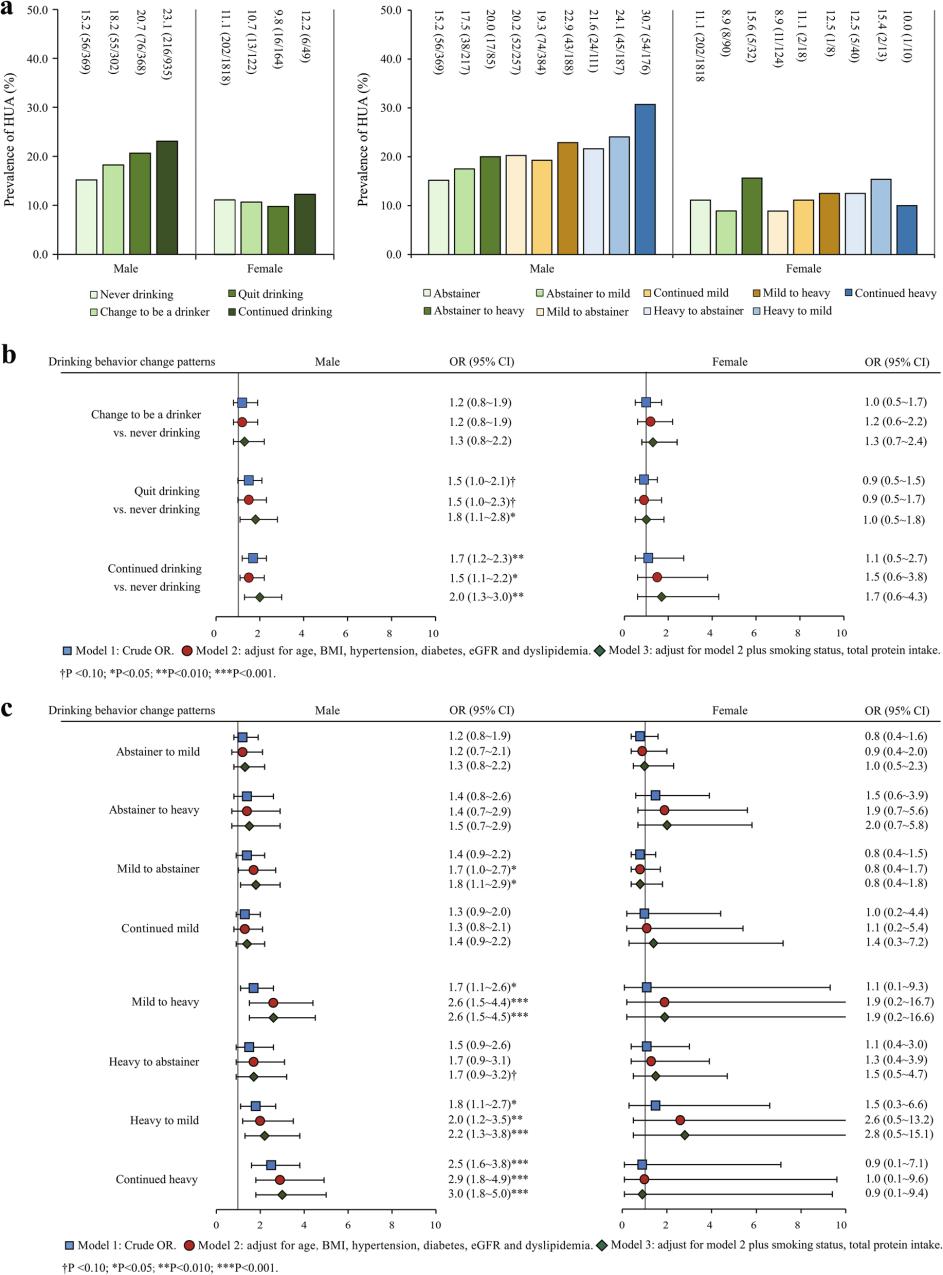
Prevalence and logistic regression analysis of the association between drinking behavior change patterns and HUA by gender (**a** Prevalence of drinking behavior change patterns; **b**/**c** Univariate and multivariate logistic regression analysis of the association between drinking behavior change patterns and HUA)

The prevalence of quitting and continued drinking in the male participants was 20.7% (76/368) and 23.1% (216/935), respectively. Further analysis showed that participants who kept heavy drinking had a higher prevalence of HUA (30.7% [54/176]) than those with the other three drinking patterns (Fig. 1a). The finding showed that quitting drinking (OR 1.5; 95% CI 1.0 ~ 2.0) and continued drinking (OR 1.7; 95% CI 1.2 ~ 2.3) was positively associated with HUA, compared to the non-drinking in male participants (Fig. 1b, model 1). After adjusting for BMI, obese WHR, diabetes, hypertension, eGFR, smoking status, and total protein intake, there was a stronger association between the drinking behaviors and HUA (adjusted odds ratio [aOR] 1.8; 95% CI 1.1 ~ 2.8; aOR 2.0; 95% CI 1.3 ~ 3.0) (Fig. 1b, model 3). Besides, mild to abstainer (aOR 1.8; 95% CI 1.1 ~ 2.9), mild to heavy (aOR 2.6; 95% CI 1.5 ~ 4.5), heavy-to-mild (aOR 2.2; 95% CI 1.3 ~ 3.8), and continued heavy drinkers (aOR 3.0; 95% CI 1.8 ~ 5.0) had higher risks of suffering from HUA (Fig. 1c, model 3).

### Correlation between early drinking behaviors and HUA

**Fig. 2:**
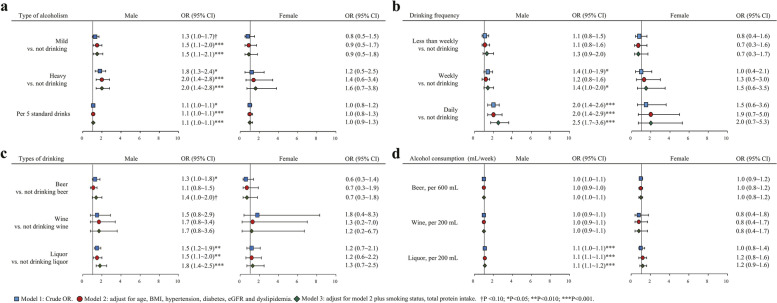
Univariate and multivariate logistic regression analysis of the association between drinking-related behaviors in 1997 and HUA by gender (OR, odds ratio; CI, confidence interval; SD, standard drink; Other abbreviations are indicated in Table 1)

Early drinking behaviors in 1997 such as mild (aOR 1.5; 95% CI 1.1 ~ 2.1) or heavy alcoholism (aOR 2.0; 95% CI 1.4 ~ 2.8), weekly alcohol drinking (aOR 1.4; 95% CI 1.0 ~ 2.0), and almost daily drinking (aOR 2.5; 95% CI 1.7 ~ 3.6) and were positively associated with HUA in the males, compared to non-drinking (Fig. 2a and b). Importantly, liquor intake was significantly associated with a higher risk of HUA (aOR 1.8; 95% CI 1.4 ~ 2.5), with 1.1 fold higher risk per 200 mL per week of liquor consumption (Fig. 2c and d). However, the association between drinking behavior and HUA were not observed in the female.

### Risk for HUA by threshold alcohol intake

**Fig. 3:**
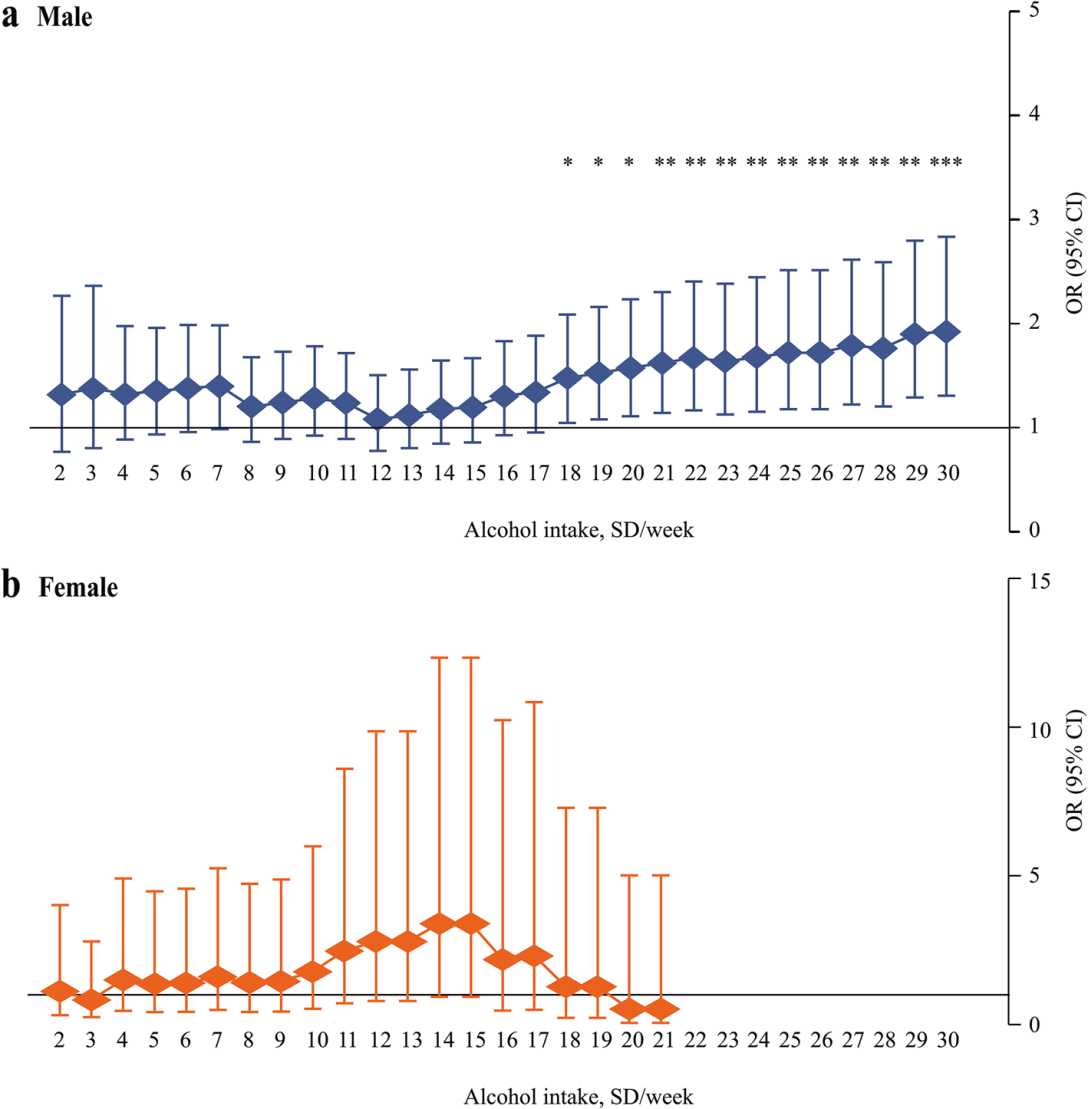
Risk of HUA by threshold alcohol intake. (OR was adjusted for age (as continuous), BMI, hypertension, diabetes, eGFR and dyslipidemia, smoking status and total protein intake; * *P* < 0.05; ** *P* < 0.010; *** *P* < 0.001; Abbreviations are indicated in Fig. 2)

We further analyzed the patterns of the threshold alcohol intake per week for HUA after adjusting for potential confounders (Fig. 3). The association of alcohol intake in 1997 with HUA was more pronounced at 18 SDs with a stable and linear increasing trend: from 1.5 times at 18 SDs to 1.9 times at 30 SDs higher risk in the male (Fig. 3a, Supplementary Table 4). In contrast, whereas the point estimates of alcohol intake per week for HUA showed a steep trend without any regularity in the females, there was no association with HUA (Fig. 3b, Supplementary Table 4).

### Explanatory analyses

As to drinking behavior change patterns, specified subgroup analyses based on gender and age (18 ~ 29, 30 ~ 44, 45 ~ 59, 60 ~ 75 years) group showed that quitting drinking (mainly manifestation for mild to abstainer) was positively associated with HUA in the 18 ~ 29 years subgroup (OR 3.0; 95% CI 1.1 ~ 8.5) in male. And continued drinking (mainly manifestation for mild to heavy, heavy to mild, and heavy to heavy) was positively associated with HUA in the 30 ~ 44, 45 ~ 59, 60 ~ 75 years subgroup (30 ~ 44 years: OR 3.4; 95% CI 1.5 ~ 7.9; 45 ~ 59 years: OR 1.9; 95% CI 1.1 ~ 3.5; 60 ~ 75 years: OR 11.3; 95% CI 1.3 ~ 97.3) in 1997, with high risk among the participants whose early drinking behaviors including heavy alcoholism, almost daily drinking and liquor intake. There existed no robust associations between drinking-related behaviors and HUA in females (Supplementary Tables 5, 6). The GEE analysis revealed significant longitudinal associations of the heavy alcoholism (OR: 1.8; 95% CI: 1.3 ~ 2.6), almost daily drinking (OR: 1.9; 95% CI: 1.3 ~ 2.7), liquor intake (OR: 1.3; 95% CI: 1.0 ~ 1.7) and liquor consumption (per 200 mL increase: OR: 1.1; 95% CI: 1.0 ~ 1.1) with a higher risk of HUA after multivariate adjustment (Supplementary Table 7).

## Discussion

The current study was the first large cohort study to explore the association between drinking behavior change patterns and HUA in the Chinese population. The findings showed that quitting drinking and continued drinking was associated with increased risks for HUA in the males, and the trends were more pronounced among those with mild to abstainer, mild to heavy, heavy to mild, and heavy to heavy drinking patterns. The magnitude of these independent associations increased further after adjusting for potential confounders. However, there was no association between the drinking patterns and HUA in the females. The rate of HUA was in sync with the estimations in our previously published meta-analysis that evaluated a whole population of 2,277,712 in China (15.5% vs. 16.4%, respectively) [4].

Although the risk of HUA was lower in those who quit drinking compared to those with continued drinking, it still elevates the risk of HUA, compared to non-drinking. This result implies that early drinking could lead to an increased risk of HUA in males. The mechanism of decreased urate excretion has been implicated in the pathogenesis of alcohol-induced HUA. The study showed that HUA develops following conversion of alcohol to lactic acid, thus reducing uric acid excretion by competitively inhibiting uric acid secretion by the proximal tubule [23]. Faller et al. report that ethanol increases urate synthesis by enhancing the turnover of adenine nucleotides [24]. In addition, ethanol administration has been shown to increase the production of uric acid by enhancing the degradation of adenosine triphosphate to adenosine monophosphate, a uric acid precursor [25]. Our findings demonstrated that current heavy drinkers (drinking in 2009) had an increased risk of HUA in male participants (Supplementary Fig. 2), early mild or heavy drinkers (drinking in 1997) had increased risks of HUA (Fig. 2). The consistent and significant association between mild to abstainer, mild to heavy drinking patterns, and HUA further validated the long-term effect of mild drinking patterns. We speculate that even mild alcohol intake could continuously decrease the glomerular filtration rate, which could promote the excretion of uric acid. Takashi et al. followed 8097 male workers for 8 years and showed that alcohol consumption at 2.5 gou/day (= ethanol 55 g/day) led to a distinct increase in the risk of HUA [26]. Baglietto et al. demonstrated that mortality curves were J-shaped (nadir at 9 ~ 12 g/day of alcohol consumption; the upper protective dose of 42 ~ 76 g/day) [27]. These findings showed that an average of 26 g/day (= 18 SD*10/7 days, Fig. 3) in 1997 or 16 g/day in 2009 (= 11 SD*10/7 days, seen in Supplementary Fig. 3) could cause a stable increase in the risk of HUA. The difference in threshold alcohol intake might be contributed to population heterogeneity (such as age, occupation, or health-related behaviors). As for the long-term effect of alcohol, our findings agreed with the Dietary Guidelines for Chinese Residents’ report which showed that adult males should drink less than 25 g of alcohol per day [28].

Consistent with a single-center study in Liaoning of China [29], our findings demonstrated that alcohol consumption increased the risk of HUA only in males rather than females. It could be explained by the fact that the sample size of female drinkers was relatively small, thus leading to a low statistical power outcome. Besides, due to differences in androgen production, the ratio of uric acid to creatinine clearance is higher in women than in men [30, 31]. There is, therefore, a need for further studies to explore the mechanism underlying our findings. Of note, distinct risks of HUA in the three types of drinking were observed in our study. Liquor drinking at baseline led to a 1.8-fold increase in the risk of HUA compared with non-liquor drinking with a 1.1-fold risk per 200 ml (Fig. 2c and d). A similar trend was observed in liquor drinkers in 2009 (Supplementary Fig. 2c and d). A 7-year cohort study (1988–1994) with 14,809 participants reported that increased SUA levels with increasing beer or liquor intake but not with increasing wine intake. However, the effect of ingested purine in beer on uric acid in blood might be sufficient to augment the HUA effect of alcohol in exerting a greater risk of gout than liquor or wine [32]. Previous studies showed that beer is the only alcoholic beverage with large purine content, which is predominantly guanosine. Guanosine is more readily absorbed than other nucleosides, nucleotides, or bases [33, 34]. Our data showed that drinking beer was marginally associated with HUA but without a dose-response relationship. Since beer contains large amounts of purines, it is feasible to speculate that the disparity in beer drinking in the male cohort could be due to the relatively small amount of beer consumption (an average of 2057 ml per week, data not shown). Because uric acid is considered an indicator for increased oxidative stress, polyphenols in wine with antioxidant properties might potentially play a role in mitigating the impact of alcohol on serum uric acid levels [35–37]. Furthermore, assessing the effect of drinking frequency in HUA showed that there was an increase in the magnitude of associations with increasing frequency of drinking [38, 39]. Thus, our findings provide a novel perspective that although the risk of HUA as a result of early drinking is lower than that associated with continued drinking, it still elevates the risk of HUA, as compared with the non-drinking.

Potential limitations of our study deserve comment. Firstly, SUA was measured only in 2009 wave in CHNS study. Therefore, drinking status at follow-up of 2009 was assessed at the same time as the assessment of HUA, leading to the weakening interpretability of the results due to the unbalanced panel data structure. Secondly, our data lacked more than half of the variables on physical activity. To bridge this gap, we tried to adjust partly for total protein intake. In addition, significant heterogeneity was observed between included follow-up and missing visits sample, which might be caused the younger age of the missing visits sample. It may produce the potential selection bias in our study. Besides, our data failed to eliminate possible effects of underlying diseases and medications used for diseases such as uric-acid-lowering medication which might have affected the outcome.

Taken together, our study demonstrated that drinking behavior change patterns such as quitting and continued drinking are strongly associated with increased risks of HUA in males. The risks emanated from early drinking behaviors such as liquor drinking, high drinking frequency, and alcohol consumption. Although the risk of HUA in quitting drinking was lower than that in continued drinking patterns, it was positively associated with HUA. The long-term effect of early drinking behaviors on HUA could not be ignored.

## Supplementary Information

**Additional file 1.**

## Data Availability

The data were available online: https://www.cpc.unc.edu/projects/ china.

## Abbreviations

BMI: BMI, body mass index
BP: blood pressure
CCDC: Chinese Center for Disease Control and Prevention
CHNS: China Health and Nutrition Survey
CKD: chronic kidney disease
CI: confidence interval
CVD: cardiovascular disease
eGFR: estimated glomerular filtration rate
ESRD: end-stage renal disease
HUA: hyperuricemia
IQR: interquartile range
MI: myocardial infarction
OR: odds ratio
SD: standard deviation
SDs: standard drinks
SUA: serum uric acid
WHR: waist to hip circumference ratio
