# Correction: Long-term drinking behavior change patterns and its association with hyperuricemia in chinese adults: evidence from China Health and Nutrition Survey

**DOI:** 10.1186/s12889-022-13794-6

**Published:** 2022-07-26

**Authors:** Bowen Zhu, Yang Li, Yiqin Shi, Nana Song, Yi Fang, Xiaoqiang Ding

**Affiliations:** 1 Department of Nephrology, Zhongshan Hospital, Fudan University, No.180 Fenglin Road, Shanghai, 200032 Shanghai China; 2 Shanghai Medical Center of Kidney, Shanghai, China; 3 Shanghai Key Laboratory of Kidney and Blood Purifcation, Shanghai, China

**Correction: BMC Public Health 22, 1230 (2022)**

**https://doi.org/10.1186/s12889-022-13637-4**

The original publication of this article [1] was missing the corresponding contact address of author Fang Yi. The contact address has been included in this correction article, the original article has been updated.

## References

[1] Zhu B (2022). Long-term drinking behavior change patterns and its association with hyperuricemia in chinese adults: evidence from China Health and Nutrition Survey. BMC Public Health.

