# Supplementary material for: Long-term drinking behavior change patterns and its association with hyperuricemia in chinese adults: evidence from China Health and Nutrition Survey
Source: BMC Public Health. 2022 Jun 20;22:1230. doi: 10.1186/s12889-022-13637-4 (PMC9210654; doi:10.1186/s12889-022-13637-4)
Supplement: Supplementary file 1 — Additional file 1. [file 12889_2022_13637_MOESM1_ESM.docx]

**Supplementary Information**

**Supplementary Table 1.** Characteristics of participants included in the analysis and those excluded from the final analysis

**Supplementary Table 2.** Characteristics of participants included follow-up sample and missing visits sample

**Supplementary Table 3.** Univariate and multivariate logistic regression analysis of the association with HUA

**Supplementary Table 4.** Risk of HUA by threshold early alcohol intake by gender

**Supplementary Table 5.** Univariate and multivariate logistic regression analysis of the association between drinking behavior change patterns and HUA by gender and age

**Supplementary Table 6.** Univariate and multivariate logistic regression analysis of the association between drinking-related behaviors in 1997 and HUA by gender and age

**Supplementary Table 7.** Results of generalized estimating equation analysis to assess the associations of drinking-related behaviors with HUA

**Supplementary Figure 1.** Flowchart showing the selection of study population

**Supplementary Figure 2.** Univariate and multivariate logistic regression analysis of the association between drinking-related behavior and HUA by gender in 2009

Supplementary Table 1. Characteristics of participants included in the analysis and those excluded from the final analysis

|  | Analytic Sample | Missing sample | *P-value** |
| --- | --- | --- | --- |
| Participants (n) | 4127 | 93 |  |
| Age (years) | 54 (±11) | 52 (±12) | 0.100 |
| Male (%) | 1974 (47.8) | 38 (40.9) | 0.183 |
| Education (years) |  |  | 0.859 |
| Never | 635 (15.4) | 17 (18.3) |  |
| ≤6 | 1425 (34.5) | 30 (32.3) |  |
| 7–9 | 1330 (32.1) | 32 (34.4) |  |
| 10–12 | 430 (10.4) | 9 (9.7) |  |
| >12 | 307 (7.4) | 5 (5.4) |  |
| Rural (%) | 2991 (72.5) | 71 (76.3) | 0.408 |
| *Anthropometry parameters* |  |  |  |
| Waist (cm) | 83 (±10) | 81 (±10) | 0.029 |
| Hip (cm) | 94 (±8) | 93 (±7) | 0.362 |
| Obese WHR | 2022 (50.6) | 38 (42.7) | 0.139 |
| BMI (kg/m2) |  |  | 0.769 |
| Lean (<18.5) | 218 (5.3) | 7 (7.5) |  |
| Normal (18.5–23.9) | 2267 (54.9) | 50 (53.8) |  |
| Overweight (24–27.9) | 1267 (30.7) | 29 (31.2) |  |
| Obesity (≥28) | 375 (9.1) | 7 (7.5) |  |
| Systolic BP (mm Hg) | 125 (±19) | 126 (±18) | 0.880 |
| Diastolic BP (mm Hg) | 81 (±11) | 81 (±10) | 0.941 |
| Hypertension | 1036 (29.3) | 26 (31.3) | 0.689 |
| Diabetes | 105 (2.5) | 7 (7.5) | 0.009 |
| Serum uric acid (mg/dL) | 5 (±2) | 5 (±2) | 0.180 |
| Hyperuricemia | 640 (15.5) | 16 (17.2) | 0.655 |
| Dyslipidemia | 2496 (60.5) | 61 (65.6) | 0.319 |
| eGFR (ml/ min/l.73m2) | 81 (±18) | 81 (±14) | 0.975 |
| History of MI | 40 (1.0) | 0 (0.0) | 0.181 |
| History of apoplexy | 41 (1.0) | 1 (1.1) | 0.936 |
| *health-related behavior* |  |  |  |
| Smoking status |  |  | 0.500 |
| Never | 2781 (67.4) | 62 (67.4) |  |
| Ever | 131 (3.2) | 1 (1.1) |  |
| Current | 1214 (29.4) | 29 (31.5) |  |
| Tea intake | 1492 (36.2) | 35 (37.6) | 0.131 |
| Coffee intake | 66 (1.6) | 1 (1.1) | 0.696 |
| Total protein intake (g/day) | 63 (±19) | 61 (±17) | 0.221 |

Abbreviation: BMI, body mass index; BP, blood pressure; eGFR, estimated glomerular filtration rate; MI, myocardial infarction; SUA, serum uric acid; WHR, waist to hip circumference ratio.

Data are presented as No. (%), mean± SD or median (IQR).

*P values were calculated by using T test for continuous variables and χ^2^ test or Fisher's exact test for categorical variables. 137 participants were not available for WHR; 601 participants were not available for hypertension; 10 participants were not available for coffee intake; 8 participants were not available for tea intake; 3 participants were not available for the history of myocardial infarction; 1 participant was not available for the history of apoplexy; 2 participants were not available for smoking status.

Supplementary Table 2. Characteristics of participants included follow-up sample and missing visits sample

|  | Follow-up Sample | Missing sample | *P-value** |
| --- | --- | --- | --- |
| Participants (n) | 5335 | 8717 |  |
| Age (years) |  |  | <0.001 |
| < 18 | 517 (9.7) | 1642 (18.8) |  |
| 18~29 | 720 (13.5) | 2601 (29.8) |  |
| 30~44 | 1907 (35.8) | 2197 (25.2) |  |
| 45~59 | 1584 (29.7) | 1560 (17.9) |  |
| 60~74 | 579 (10.9) | 584 (6.7) |  |
| ≥ 75 | 28 (0.5) | 133 (1.5) |  |
| Male (%) | 2667 (50.0) | 4254 (48.8) | 0.171 |
| Education (years) |  |  | <0.001 |
| Never | 867 (16.3) | 1166 (15.0) |  |
| ≤6 | 1672 (31.4) | 3012 (38.8) |  |
| 7–9 | 1763 (33.1) | 2441 (31.4) |  |
| 10–12 | 560 (10.5) | 783 (10.1) |  |
| >12 | 461 (8.7) | 369 (4.8) |  |
| Rural (%) | 3806 (71.3) | 6244 (71.6) | 0.712 |
| *Anthropometry parameters* |  |  |  |
| Waist (cm) | 82.0 ±10.6 | 71.3 ±12.1 | <0.001 |
| Hip (cm) | 93.7 ±8.0 | 83.7 ±12.9 | <0.001 |
| Obese WHR | 2474 (48.0) | 2399 (35.8) | <0.001 |
| BMI (kg/m2) |  |  | <0.001 |
| Lean (<18.5) | 432 (8.1) | 2074 (23.8) |  |
| Normal (18.5–23.9) | 2958 (55.5) | 5613 (64.4) |  |
| Overweight (24–27.9) | 1492 (28.0) | 866 (9.9) |  |
| Obesity (≥28) | 453 (8.5) | 164 (1.9) |  |
| Systolic BP (mm Hg) | 125.1 ±19.4 | 112.5 ±19.3 | <0.001 |
| Diastolic BP (mm Hg) | 80.2 ±11.3 | 72.8 ±11.7 | <0.001 |
| Hypertension | 1331 (28.9) | 654 (9.8) | <0.001 |
| Diabetes | 131 (2.5) | 106 (1.2) | <0.001 |
| History of MI | 53 (1.0) | 11 (0.2) | 0.181 |
| History of apoplexy | 58 (1.1) | 35 (0.5) | 0.936 |
| *health-related behavior* |  |  |  |
| Smoking status |  |  | <0.001 |
| Never | 3632 (68.1) | 4941 (70.2) |  |
| Ever | 178 (3.3) | 104 (1.5) |  |
| Current | 1523 (28.6) | 1989 (28.3) |  |
| Drinking status |  |  |  |
| Never | 3551 (66.6) | 4679 (67.7) | 0.386 |
| Ever | 177 (3.3) | 213 (3.1) |  |
| Current | 1605 (30.1) | 2018 (29.2) |  |
| Tea intake | 1893 (35.5) | 3386 (48.8) | <0.001 |
| Coffee intake | 106 (2.0) | 39 (0.6) | <0.001 |
| Total protein intake (g/day) | 65.4 ±23 | 64.9 ±25.4 | 0.228 |

Abbreviation: BMI, body mass index; BP, blood pressure; MI, myocardial infarction; SUA, serum uric acid; WHR, waist to hip circumference ratio. Data are presented as No. (%), mean± SD.

*P values were calculated by using T test for continuous variables and χ^2^ test or Fisher's exact test for categorical variables. 958 participants were not available for education levels; 2197 participants were not available for WHR; 2786 participants were not available for hypertension; 1685 participants were not available for smoking status; 1809 participants were not available for drinking status; 1743 participants were not available for coffee intake; 1775 participants were not available for tea intake; 1889 participants were not available for the history of myocardial infarction; 2001 participant was not available for the history of apoplexy.

Supplementary Table 3. Univariate and multivariate logistic regression analysis of the association with HUA

|  | HUA | | *P-value** | Unadjusted OR  (95% CI) | *P-value* |  | Multivariable  adjusted OR  (95% CI) | P*-value* |
| --- | --- | --- | --- | --- | --- | --- | --- | --- |
|  | No | Yes |  |  |  |  |  |  |
| Participants (n) | 3487 | 640 |  |  |  |  |  |  |
| Age, per 5 year increase | 54 (±11) | 55 (±11) | 0.047 | 1.0 (1.0~1.1) | 0.047 |  | 0.9 (0.9~1.0) | 0.012 |
| Male (%) | 1571 (45.1) | 403 (63.0) | <0.001 | 2.1 (1.7~2.5) | <0.001 |  | 1.7 (1.2~2.3) | 0.003 |
| Education (years) |  |  | 0.001 |  | 0.051 |  |  |  |
| 0 | 551 (15.8) | 84 (13.2) |  | 0.9 (0.7~1.2) | 0.703 |  | 1.2 (0.9~1.6) | 0.321 |
| ≤6 | 1228 (35.2) | 197 (30.8) |  | Ref (1.00) | |  | Ref (1.00) | |
| 7–9 | 1111 (31.8) | 219 (34.3) |  | 1.2 (1.0~1.5) | 0.053 |  | 1.2 (0.9~1.6) | 0.142 |
| 10–12 | 360 (10.3) | 70 (11.0) |  | 1.2 (0.9~1.6) | 0.210 |  | 1.0 (0.7~1.5) | 0.982 |
| >12 | 238 (6.8) | 69 (10.8) |  | 1.8 (1.3~2.5) | <0.001 |  | 1.2 (0.8~1.9) | 0.313 |
| Urban | 926 (26.6) | 210 (32.8) | 0.001 | 1.4 (1.1~1.6) | <0.001 |  | 1.3 (1.0~1.6) | 0.023 |
| *Anthropometry parameters* |  |  |  |  |  |  |  |  |
| Obese WHR | 1632 (48.4) | 390 (62.9) | <0.001 | 1.8 (1.5~2.2) | <0.001 |  | 1.4 (1.1~1.7) | 0.006 |
| BMI (kg/m2) |  |  | <0.001 |  | <0.001 |  |  |  |
| Lean (<18.5) | 206 (5.9) | 12 (1.9) |  | 0.5 (0.3~0.8) | 0.010 |  | 0.7 (0.3~1.3) | 0.208 |
| Normal (18.5–23.9) | 2011 (57.7) | 256 (40.0) |  | Ref (1.00) | |  | Ref (1.00) | |
| Overweight (24–27.9) | 1003 (28.8) | 264 (41.3) |  | 2.1 (1.7~2.5) | <0.001 |  | 0.7 (0.3~1.3) | <0.001 |
| Obesity (≥28) | 267 (7.7) | 108 (16.9) |  | 3.2 (2.5~4.1) | <0.001 |  | 1.6 (1.3~2.1) | <0.001 |
| Hypertension | 796 (26.7) | 240 (43.3) | <0.001 | 2.1 (1.7~2.5) | <0.001 |  | 1.4 (1.1~1.8) | 0.002 |
| Diabetes | 79 (2.3) | 26 (4.1) | 0.008 | 1.8 (1.2~2.9) | 0.009 |  | 0.9 (0.5~1.6) | 0.648 |
| Dyslipidemia | 1959 (56.2) | 537 (83.9) | <0.001 | 4.1 (3.3~5.1) | <0.001 |  | 2.9 (2.2~3.7) | <0.001 |
| History of MI | 30 (0.9) | 10 (1.6) | 0.096 | 1.8 (0.9~3.8) | 0.101 |  |  |  |
| History of apoplexy | 32 (0.9) | 9 (1.4) | 0.252 | 1.5 (0.7~3.2) | 0.255 |  |  |  |
| Framingham score (%)，per 10 unit increase | 3.9 (2.0~7.3) | 5.6 (3.3~10.0) | <0.001 | 1.6 (1.4~1.7) | <0.001 |  | 1.1 (0.8~1.4) | 0.601 |
| eGFR, per 10 unit increase | 77 (±15) | 69 (±17) | <0.001 | 0.6 (0.6~0.7) | <0.001 |  | 0.6 (0.5~0.6) | <0.001 |
| *Health-related behaviour* |  |  |  |  |  |  |  |  |
| Smoking status |  |  | <0.001 |  | <0.001 |  |  |  |
| Never | 2391 (68.6) | 390 (60.9) |  | Ref (1.00) | |  | Ref (1.00) | |
| Ever | 104 (3.0) | 27 (4.2) |  | 1.6 (1.0~2.5) | 0.037 |  | 0.8 (0.5~1.5) | 0.533 |
| Current | 991 (28.4) | 223 (34.8) |  | 1.4 (1.2~1.7) | <0.001 |  | 1.1 (0.8~1.4) | 0.647 |
| Tea intake | 1224 (35.1) | 268 (41.9) | 0.005 | 1.3 (1.1~1.6) | 0.001 |  | 0.9 (0.7~1.1) | 0.229 |
| Coffee intake | 51 (1.5) | 15 (2.4) | 0.103 | 1.6 (0.9~2.9) | 0.106 |  |  |  |
| Total protein intake, per 10 g increase | 62 (±19) | 65 (±19) | <0.001 | 1.1 (1.0~1.1) | <0.001 |  | 1.1 (1.0~1.1) | 0.031 |

| Physical activity level (METs/week) |  |  | 0.778 |  |  |  |  |  |
| --- | --- | --- | --- | --- | --- | --- | --- | --- |
| Low (<49.6) | 1161 (33.3) | 215 (33.6) |  |  |  |  |  |  |
| Medium (49.6~143.7) | 1156 (33.2) | 219 (34.2) |  | 1 (0.8~1.3) | 0.828 |  |  |  |
| High (>143.7) | 1170 (33.6) | 206 (32.2) |  | 1 (0.8~1.2) | 0.634 |  |  |  |
| Drinking behavior change pattern |  |  | <0.001 |  | <0.001 |  |  |  |
| Never drinking | 1929 (55.3) | 258 (40.3) |  | Ref (1.00) | |  | Ref (1.00) | |
| Change to be a drinker | 356 (10.2) | 68 (10.6) |  | 1.4 (1.1~1.9) | 0.016 |  | 1.2 (0.8~1.8) | 0.306 |
| Quit drinking | 440 (12.6) | 92 (14.4) |  | 1.6 (1.2~2.0) | <0.001 |  | 1.3 (0.9~1.8) | 0.177 |
| Keep drinking | 762 (21.9) | 222 (34.7) |  | 2.2 (1.8~2.7) | <0.001 |  | 1.7 (1.2~2.4) | 0.002 |

Abbreviation: OR, odds ratio; CI, confidence interval; Other abbreviations as in Supplementary table 1. Abbreviations as in Table 1; Data are presented as No. (%), mean± SD or median (IQR);

*P values were calculated by using T test or Wilcoxon test for continuous variables and χ^2^ test for categorical variables.

133 participants were not available for WHR; 591 participants were not available for hypertension; 31 participants were not available for drinking frequency; 9 participants were not available for coffee intake; 7 participants were not available for tea intake; 3 participants were not available for the history of myocardial infarction; 1 participant was not available for the history of apoplexy; 1 participant was not available for smoking status; 26 participants were not available for drinking frequency; 9 participants were not available for beer drinking; 10 participants were not available for wine drinking; 11 participants were not available for liquor drinking.

Supplementary Table 4. Risk of HUA by threshold early alcohol intake by gender

| Threshold alcohol intake, SD/week | Male |  | Female |
| --- | --- | --- | --- |
|  | Multivariable  adjusted OR (95% CI) |  | Multivariable  adjusted OR (95% CI) |
| 1 | N.A |  | N.A |
| 2 | 1.3 (0.8~2.3) |  | 1.1 (0.3~4.0) |
| 3 | 1.4 (0.8~2.4) |  | 0.8 (0.2~2.8) |
| 4 | 1.3 (0.9~2.0) |  | 1.5 (0.5~4.9) |
| 5 | 1.4 (0.9~2.0) |  | 1.4 (0.4~4.5) |
| 6 | 1.4 (1.0~2.0)^†^ |  | 1.4 (0.4~4.6) |
| 7 | 1.4 (1.0~2.0)^†^ |  | 1.6 (0.5~5.3) |
| 8 | 1.2 (0.9~1.7) |  | 1.4 (0.4~4.7) |
| 9 | 1.2 (0.9~1.7) |  | 1.5 (0.4~4.9) |
| 10 | 1.3 (0.9~1.8) |  | 1.8 (0.5~6.0) |
| 11 | 1.2 (0.9~1.7) |  | 2.5 (0.7~8.6) |
| 12 | 1.1 (0.8~1.5) |  | 2.8 (0.8~9.9) |
| 13 | 1.1 (0.8~1.6) |  | 2.8 (0.8~9.9) |
| 14 | 1.2 (0.8~1.6) |  | 3.4 (0.9~12.3)^†^ |
| 15 | 1.2 (0.9~1.7) |  | 3.4 (0.9~12.3)^†^ |
| 16 | 1.3 (0.9~1.8) |  | 2.2 (0.5~10.2) |
| 17 | 1.3 (1.0~1.9)^†^ |  | 2.3 (0.5~10.8) |
| 18 | 1.5 (1.0~2.1)^*^ |  | 1.3 (0.2~7.3) |
| 19 | 1.5 (1.1~2.2)^*^ |  | 1.3 (0.2~7.3) |
| 20 | 1.6 (1.1~2.2)^*^ |  | 0.5 (0.1~5.0) |
| 21 | 1.6 (1.1~2.3)^**^ |  | 0.5 (0.1~5.0) |
| 22 | 1.7 (1.2~2.4)^**^ |  | N.A |
| 23 | 1.6 (1.1~2.4)^**^ |  | N.A |
| 24 | 1.7 (1.2~2.4)^**^ |  | N.A |
| 25 | 1.7 (1.2~2.5)^**^ |  | N.A |
| 26 | 1.7 (1.2~2.5)^**^ |  | N.A |
| 27 | 1.8 (1.2~2.6)^**^ |  | N.A |
| 28 | 1.8 (1.2~2.6)^**^ |  | N.A |
| 29 | 1.9 (1.3~2.8)^**^ |  | N.A |
| 30 | 1.9 (1.3~2.8)^***^ |  | N.A |

OR was adjusted for age (as continous), gender, BMI, HTN, diabetes, eGFR and dyslipidemia, smoking status and total protein intake; †P <0.10; * P<0.05; ** P<0.010; *** P<0.001; Abbreviations as in Supplementary table 1.

Supplementary Table 5. Univariate and multivariate logistic regression analysis of the association between drinking behavior change patterns and HUA by gender and age

|  | Multivariable-adjusted OR (95% CI) | | | |
| --- | --- | --- | --- | --- |
|  | 18~29 | 30~44 | 45~59 | 60~75 |
| ***Male*** |  |  |  |  |
| Drinking behavior change pattern1 |  |  |  |  |
| Never drinking | 1.00(Ref) | 1.00(Ref) | 1.00(Ref) | 1.00(Ref) |
| Change to drinking | 1.3 (0.5~3.4) | 1.7 (0.7~4.6) | 1.1 (0.5~2.5) | 17.2 (0.3~880.3) |
| Quitting drinking | 3.0 (1.1~8.5)* | 2.4 (0.9~6.2) | 1.5 (0.7~2.9) | 3.0 (0.3~29.5) |
| Continued drinking | 0.8 (0.3~2.1) | 3.4 (1.5~7.9)* | 1.9 (1.1~3.5)* | 11.3 (1.3~97.2)* |
| Drinking behavior change pattern2 |  |  |  |  |
| Never drinkig, | 1.00(Ref) | 1.00(Ref) | 1.00(Ref) | 1.00(Ref) |
| Abstainer to mild, | 1.5 (0.6~3.9) | 1.7 (0.6~5.0) | 0.9 (0.3~2.4) | N.A |
| Abstainer to heavy, | 1.0 (0.3~3.8) | 2.1 (0.6~7.4) | 1.6 (0.4~5.6) | N.A |
| Mild to abstainer | 3.8 (1.3~11.4)* | 1.8 (0.6~5.2) | 1.6 (0.8~3.3) | 3.0 (0.2~41.1) |
| Mild to mild. | 1.0 (0.4~2.9) | 1.8 (0.7~4.7) | 1.5 (0.7~3.2) | 4.1 (0.2~73.4) |
| Mild to heavy | 0.9 (0.2~3.5) | 5.7 (2.1~15.6)*** | 2.4 (1.0~5.6)* | N.A |
| Heavy to abstainer, | 1.6 (0.3~8.3) | 4.2 (1.3~13.3)* | 1.2 (0.5~3.1) | 4.9 (0.2~129.6) |
| Heavy to mild | 0.2 (0.1~1.8) | 6.4 (2.3~17.3)* | 1.4 (0.6~3.4) | 26.1 (1.6~439.9)* |
| Heavy to heavy | 1.2 (0.1~11.1) | 4.0 (1.4~11.0)** | 3.0 (1.4~6.5) | N.A |
| ***Female*** |  |  |  |  |
| Drinking behavior change pattern1 |  |  |  |  |
| Never drinking | 1.00(Ref) | 1.00(Ref) | 1.00(Ref) | 1.00(Ref) |
| Change to drinking | 1.4 (0.1~14.8) | 1.6 (0.7~4.0) | 0.8 (0.2~2.9) | 2.4 (0.2~32.6) |
| Quitting drinking | N.A | 0.5 (0.1~1.7) | 1.5 (0.6~3.7) | 2.0 (0.3~15.2) |
| Continued drinking | N.A | 1.3 (0.3~6.6) | 2.5 (0.7~8.9) | N.A |
| Drinking behavior change pattern2 |  |  |  |  |
| Never drinkig, | 1.00(Ref) | 1.00(Ref) | 1.00(Ref) | 1.00(Ref) |
| Abstainer to mild, | 2.3 (0.2~27.5) | 1.6 (0.6~4.3) | N.A | 3.4 (0.2~55.3) |
| Abstainer to heavy, | N.A | 2.0 (0.3~12.3) | 2.7 (0.7~10.6) | N.A |
| Mild to abstainer | N.A | 0.2 (0.1~1.7) | 1.5 (0.6~3.9) | 1.2 (0.1~14.2) |
| Mild to mild. | N.A | 1.9 (0.2~20.2) | 2.1 (0.2~24.1) | N.A |
| Mild to heavy | N.A | 3.9 (0.4~42.1) | N.A | N.A |
| Heavy to abstainer, | N.A | 1.2 (0.3~5.6) | 1.7 (0.2~14.8) | 6.2 (0.2~209.5) |
| Heavy to mild | N.A | N.A | 7.7 (1.1~52.6)* | N.A |
| Heavy to heavy | N.A | N.A | 1.3 (0.1~18.7) | N.A |

OR, odds ratio; CI, confidence interval; OR was adjusted for age (as continuous), BMI, hypertension, diabetes, eGFR and dyslipidemia, smoking status and total protein intake; * P<0.05; ** P<0.010; *** P<0.001.

Supplementary Table 6. Univariate and multivariate logistic regression analysis of the association between drinking-related behaviors in 1997 and HUA by gender and age

|  | Multivariable-adjusted OR (95% CI) | | | |
| --- | --- | --- | --- | --- |
|  | 18~29 | 30~44 | 45~59 | 60~75 |
| ***Male*** |  |  |  |  |
| Type of alcoholism |  |  |  |  |
| No drinking | 1.00(Ref) | 1.00(Ref) | 1.00(Ref) | 1.00(Ref) |
| Mild | 1.3 (0.7~2.4) | 1.8 (1.0~3.2) | 1.6 (1.0~2.8) | 1.3 (0.2~8.6) |
| Heavy | 0.6 (0.2~1.7) | 3.2 (1.7~6.0)^***^ | 1.8 (1.0~3.2)^*^ | 9.5 (1.4~66.3)^*^ |
| Drinking frequency |  |  |  |  |
| No drinking | 1.00(Ref) | 1.00(Ref) | 1.00(Ref) | 1.00(Ref) |
| Less than weekly | 1.3 (0.6~2.8) | 1.5 (0.7~3.1) | 1.5 (0.8~2.9) | 0.2 (0.1~3.6) |
| Weekly | 0.7 (0.3~1.6) | 2.1 (1.2~3.9)^***^ | 1.5 (0.8~2.7) | 2.9 (0.2~44.6) |
| Daily | 1.8 (0.6~5.9) | 3.8 (1.9~7.4)^**^ | 2.0 (1.1~3.4)^*^ | 16.5 (2.0~137.3)^*^ |
| Types of drinking |  |  |  |  |
| Beer vs. non beer | 1.4 (0.6~2.9) | 2.1 (1.1~3.9) | 1.0 (0.5~2.0) | 2.6 (0.1~147.3) |
| Wine vs. non wine | 7.6 (0.4~158.3) | 4.9 (1.4~17.8)^**^ | 0.5 (0.1~2.4) | 1.0 (0.1~100.0) |
| Liquor vs. non liquor | 1.1 (0.5~2.2) | 2.5 (1.4~4.4)^**^ | 1.9 (1.2~3.2)^**^ | 6.1 (1.0~37.4) |
| Alcohol consumption |  |  |  |  |
| Beer, per 600 mL | 1.0 (0.9~1.1) | 1.0 (1.0~1.1) | 1.1 (1.0~1.2) | 0.7 (0.2~2.0) |
| Wine, per 200 mL | 1.1 (0.7~1.9) | 1.3 (1.1~1.6)^**^ | 0.9 (0.7~1.3) | N.A |
| Liquor, per 200 mL | 1.0 (0.8~1.2) | 1.1 (1.0~1.2)^*^ | 1.1 (1.0~1.2)^*^ | 1.3 (1.0~1.9) |
| ***Female*** |  |  |  |  |
| Type of alcoholism |  |  |  |  |
| No drinking | 1.00(Ref) | 1.00(Ref) | 1.00(Ref) | 1.00(Ref) |
| Mild | N.A | 0.6 (0.2~1.9) | 1.5 (0.6~3.6) | 1.0 (0.1~11.0) |
| Heavy | N.A | 0.8 (0.2~3.7) | 2.8 (0.8~10) | 4.4 (0.2~81.9) |
| Drinking frequency |  |  |  |  |
| No drinking | 1.00(Ref) | 1.00(Ref) | 1.00(Ref) | 1.00(Ref) |
| Less than weekly | N.A | N.A | 1.7 (0.6~5.0) | 1.4 (0.1~17.3) |
| Weekly | N.A | 1.5 (0.4~5.6) | 1.2 (0.3~4.5) | 3.2 (0.2~63.1) |
| Daily | N.A | 1.5 (0.3~7.3) | 4.4 (1.1~18.6)^*^ | N.A |
| Types of drinking |  |  |  |  |
| Beer vs. non beer | N.A | 0.3 (0.1~2.1) | 1.6 (0.5~5.3) | N.A |
| Wine vs. non wine | N.A | N.A | 3.9 (0.4~35.3) | N.A |
| Liquor vs. non liquor | N.A | 1.0 (0.3~3.0) | 1.6 (0.6~4.0) | 5.1 (0.5~48.8) |
| Alcohol consumption |  |  |  |  |
| Beer, per 600 mL | N.A | 1.1 (0.7~1.6) | 1.0 (0.5~1.9) | N.A |
| Wine, per 200 mL | N.A | 0.6 (0.1~5.0) | 2.4 (0.6~10.6) | 2.7 (0.4~16.1) |
| Liquor, per 200 mL | N.A | 1.5 (1.0~2.3) | 1.4 (1.0~2.0) | N.A |

OR, odds ratio; CI, confidence interval; OR was adjusted for age (as continuous), BMI, hypertension, diabetes, eGFR and dyslipidemia, smoking status and total protein intake; * P<0.05; ** P<0.010; *** P<0.001.

Supplementary Table 7. Results of generalized estimating equation analysis to assess the associations of drinking-related behaviors with HUA

|  | Model 1 |  | Model 2 |  | Model 3 |
| --- | --- | --- | --- | --- | --- |
|  | OR (95% CI) |  | OR (95% CI) |  | OR (95% CI) |
| ***Male*** |  |  |  |  |  |
| Alcohol drinking |  |  |  |  |  |
| No | 1.00(Ref) |  | 1.00(Ref) |  | 1.00(Ref) |
| Yes | 1.2 (0.9~1.5) |  | 1.2 (0.8~1.7) |  | 1.2 (0.8~1.7) |
| Type of alcoholism |  |  |  |  |  |
| No drinking | 1.00(Ref) |  | 1.00(Ref) |  | 1.00(Ref) |
| Mild | 1.1 (0.9~1.5) |  | 1.1 (0.8~1.5) |  | 1.1 (0.8~1.6) |
| Heavy | 1.6 (1.2~2.1)^**^ |  | 1.9 (1.3~2.6)^***^ |  | 1.8 (1.3~2.6)^**^ |
| Drinking frequency |  |  |  |  |  |
| No drinking | 1.00(Ref) |  | 1.00(Ref) |  | 1.00(Ref) |
| Less than weekly | 0.9 (0.6~1.3) |  | 0.9 (0.6~1.3) |  | 0.9 (0.6~1.4) |
| Weekly | 1.4 (1.0~1.8)^*^ |  | 1.4 (0.9~1.9) |  | 1.3 (0.9~1.9) |
| Daily | 1.6 (1.2~2.2)^***^ |  | 1.9 (1.4~2.7)^***^ |  | 1.9 (1.3~2.7)^***^ |
| Types of drinking |  |  |  |  |  |
| Beer vs. non beer | 0.9 (0.7~1.2) |  | 0.9 (0.7~1.2) |  | 0.9 (0.7~1.2) |
| Wine vs. non wine | 1.4 (0.9~2.1) |  | 1.2 (0.8~2.0) |  | 1.2 (0.8~1.9) |
| Liquor vs. non liquor | 1.2 (1.0~1.5) |  | 1.4 (1.0~1.8)^*^ |  | 1.3 (1.0~1.7)^*^ |
| Alcohol consumption |  |  |  |  |  |
| Beer, per 600 mL | 1.0 (1.0~1.1) |  | 1.0 (1.0~1.1) |  | 1.0 (1.0~1.1) |
| Wine, per 200 mL | 1.1 (1.0~1.3)^*^ |  | 1.2 (1.0~1.3)^*^ |  | 1.2 (1.0~1.3)^*^ |
| Liquor, per 200 mL | 1.1 (1.0~1.1)^***^ |  | 1.1 (1.0~1.1)^***^ |  | 1.1 (1.0~1.1)^***^ |
| ***Female*** |  |  |  |  |  |
| Alcohol drinking |  |  |  |  |  |
| No | 1.00(Ref) |  | 1.00(Ref) |  | 1.00(Ref) |
| Yes | 1.3 (0.5~3.5) |  | 1.8 (0.5~5.8) |  | 1.9 (0.6~6.7) |
| Type of alcoholism |  |  |  |  |  |
| No drinking | 1.00(Ref) |  | 1.00(Ref) |  | 1.00(Ref) |
| Mild | 0.9 (0.5~1.6) |  | 1.1 (0.5~2.2) |  | 1.2 (0.6~2.5) |
| Heavy | 1.3 (0.6~3.0) |  | 1.7 (0.7~4.5) |  | 1.7 (0.7~4.6) |
| Drinking frequency |  |  |  |  |  |
| No drinking | 1.00(Ref) |  | 1.00(Ref) |  | 1.00(Ref) |
| Less than weekly | 1.0 (0.5~2.1) |  | 1.2 (0.5~2.6) |  | 1.3 (0.6~3.0) |
| Weekly | 1.2 (0.5~2.7) |  | 2.6 (1.0~6.6)^*^ |  | 2.8 (1.1~7.3)^*^ |
| Daily | 0.9 (0.3~3.0) |  | 0.7 (0.2~2.5) |  | 0.7 (0.2~2.6) |
| Types of drinking |  |  |  |  |  |
| Beer vs. non beer | 1.0 (0.5~2.0) |  | 1.6 (0.7~3.4) |  | 1.8 (0.8~3.8) |
| Wine vs. non wine | 1.2 (0.5~2.8) |  | 1.7 (0.6~4.7) |  | 1.8 (0.6~5.1) |
| Liquor vs. non liquor | 1.0 (0.5~1.9) |  | 1.3 (0.6~2.8) |  | 1.4 (0.6~3.0) |
| Alcohol consumption |  |  |  |  |  |
| Beer, per 600 mL | 0.9 (0.6~1.2) |  | 1.0 (0.7~1.5) |  | 1.0 (0.7~1.4) |
| Wine, per 200 mL | 1.1 (0.8~1.5) |  | 1.2 (0.7~1.9) |  | 1.2 (0.8~1.9) |
| Liquor, per 200 mL | 1.2 (1.0~1.5) |  | 1.3 (1.1~1.7)^*^ |  | 1.3 (1.1~1.7)^*^ |

OR, odds ratio; CI, confidence interval; Model 1: Unadjust OR; Model 2: Adjust for age (as continous), BMI, HTN, diabetes, eGFR and dyslipidemia; Model 3: Adjust for model 2 plus smoking status and total protein intake. * P<0.05; ** P<0.010; *** P<0.001


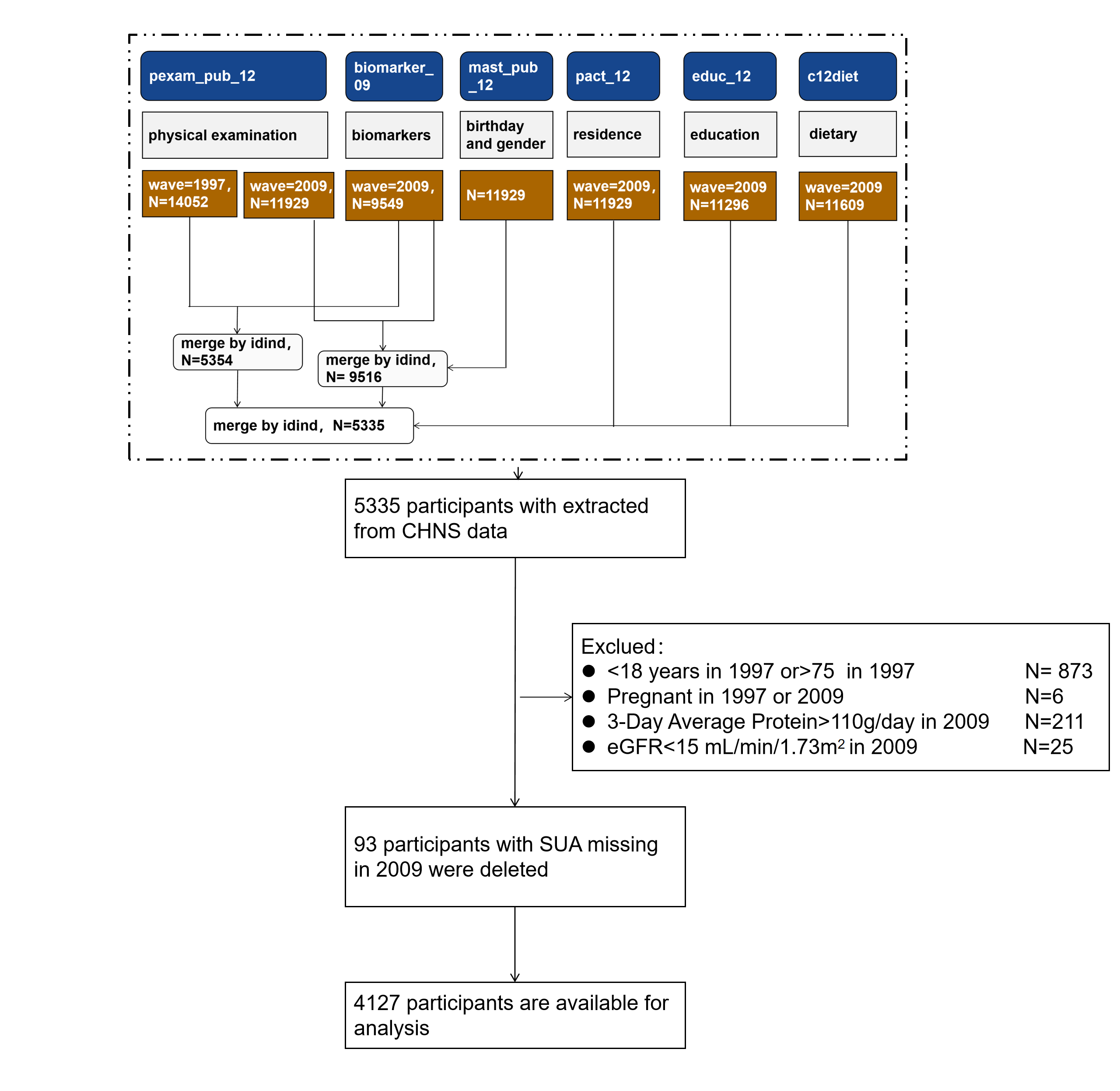


Supplementary Figure 1. Flowchart showing the selection of study population


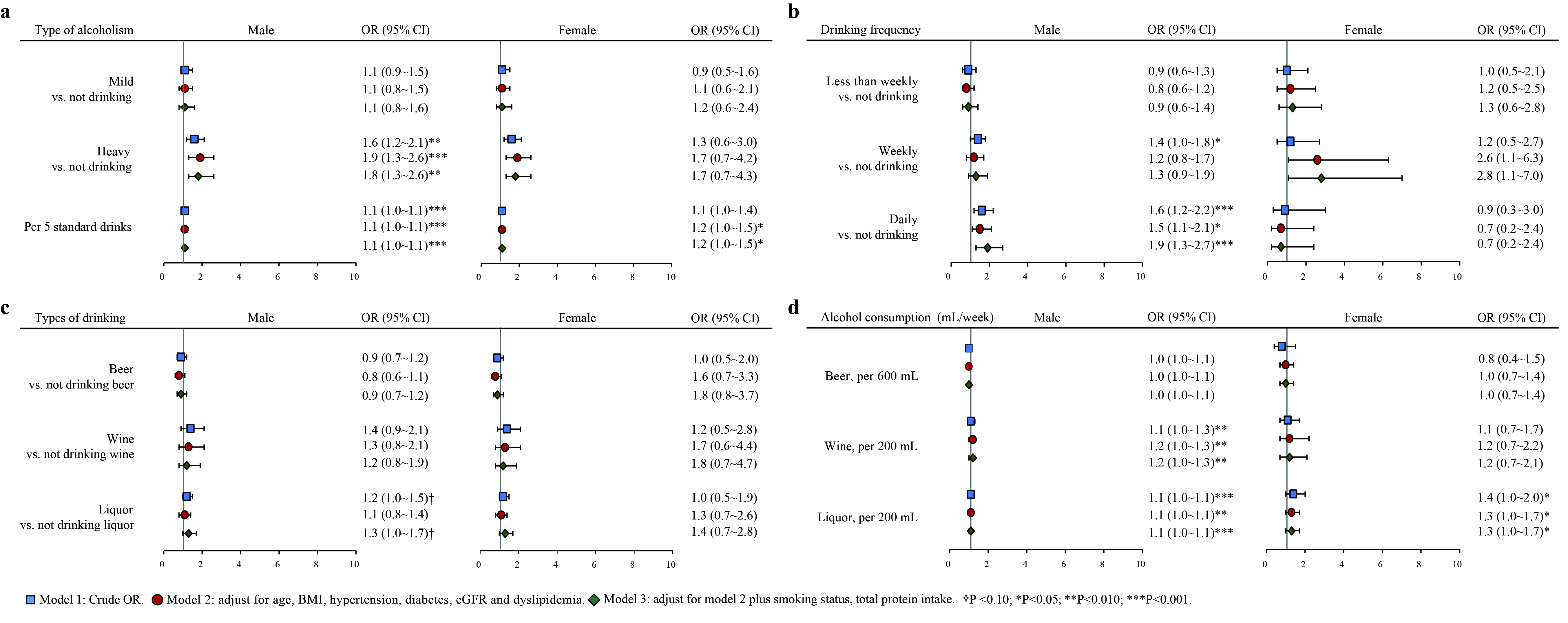


Supplementary Figure 2. Univariate and multivariate logistic regression analysis of the association between drinking-related behavior and HUA by gender in 2009 (OR, odds ratio; CI, confidence interval; SD, standard drink; Other abbreviations as in Table 1).
